# Risk factors and long-term outcomes of acute kidney injury complication after type A acute aortic dissection surgery in young patients

**DOI:** 10.1186/s13019-020-01365-y

**Published:** 2020-10-15

**Authors:** Qiuyan Zong, Min Ge, Tao Chen, Cheng Chen, Zhigang Wang, Dongjin Wang

**Affiliations:** Department of Cardio-Thoracic Surgery, Nanjing Drum Tower Hospital, The Affiliated Hospital of Nanjing University Medical School, Nanjing, 210008 China

**Keywords:** Acute kidney injury, Type A aortic dissection, Risk factors, Young age, Cystatin C

## Abstract

**Objective:**

To identify risk factors and long-term outcomes of acute kidney injury (AKI) in young patients who underwent type A acute aortic dissection (TA-AAD) emergency surgeries.

**Methods:**

This retrospective study enrolled 121 consecutive patients less than 40 years old who received TA-AAD emergency surgeries between January 2014 to December 2018 in Nanjing Drum Tower hospital. The diagnosis of AKI was made based on the KDIGO criteria. Multivariable regression analysis was performed to identify risk factors for postoperative AKI. Kaplan–Meier curves were generated to compare long-term outcomes between patients with and without AKI complication after TA-AAD surgeries.

**Results:**

Among all enrolled patients, AKI occurred in 51 patients (42.1%) and renal replacement therapy (RRT) was required in 15 patients (12.4%). The development of postoperative AKI was associated with increased 30-day mortality (*P* = 0.041), longer ICU stay time (*P* < 0.001) and hospital stay time (*P* = 0.006). Multivariable analysis indicated that elevated preoperative serum cystatin C (sCyC) (OR = 6.506, 95% CI: 1.852–22.855, *P* = 0.003) was the only independent risk factor for developing AKI. The areas under the receiver-operating characteristic curve (AUC) of preoperative sCyC was 0.800 (95% CI: 0.719, 0.882). Preoperative sCyC had a sensitivity of 64.7% and a specificity of 83.8% in diagnosing postoperative AKI with a cut-off value of 0.895 mg/L. In addition, our data suggested there was no difference discovered regarding long-term cumulative survival rate between patients with and without AKI during a median 29 months follow-up period.

**Conclusions:**

Postoperative AKI after TA-AAD surgeries was relatively common in young patients and associated with increased short-term mortality. Elevated preoperative sCyC was identified as an independent risk factor for AKI with potential diagnostic merit.

## Introduction

Aortic dissection (AD) is a life-threatening disease. Despite recent advances in clinical management and surgical techniques, type A acute aortic dissection (TA-AAD) still carries a high burden of morbidity and mortality [1]. Acute kidney injury (AKI) is a relative common serious complication after TA-AAD repair surgery. It had been proved as an independent risk factor for disease prognosis in patients received TA-AAD surgeries [2]. Early identification of AKI in postoperative period allow timely therapeutic intervention to prevent disease progression and improve prognosis [3].

AD is relatively uncommon in young patients and it has been suggested only 6.4% of all thoracic aortic dissections occurred in patients under 40 years old [4]. Some reports have been published characterizing young patients with aortic dissection [4–6]. However, the incidence and long-term outcome of AKI in young patients who received extended aortic repairments have been not well studied. The primary aims of this study were to identify the incidence and risk factors of AKI in young patients who received surgical treatment for TA-AAD and to investigate its impact on both short- and long-term outcomes.

## Methods

The ethics committee of Nanjing Drum Tower hospital approved this retrospective study and waived the need for individual informed consent due to the retrospective nature of this study.

### Patients

The medical records of patients (age < 40) who received emergency TA-AAD surgery between January 2014 and December 2018 were reviewed retrospectively. Patients with history of preoperative renal replacement therapy (RRT) and died within 24 h postoperatively were excluded. After screening, a total of 121 consecutive patients were enrolled for the present study.

### Diagnostic criteria for postoperative AKI

The postoperative AKI was diagnosed according to the Kidney Disease Improving Global Outcomes (KDIGO) criteria [7]. The AKI criteria comprised of an absolute increase in serum creatinine (sCr) of more than or equal to 0.3 mg/dL or a percentage increase in the sCr of more than or equal to 50% up to 7 days after surgery.

### Surgical procedures

TA-AAD surgeries were performed following standard procedures. Specifically, after systemic heparinization, cardiopulmonary bypass (CPB) was established by cannulation of femoral artery or right axillary artery and atrium. When patients were cooled to 18 °C—20 °C (nasopharyngeal temperature), the circulation was stopped, and the flow rate was reduced to 3–5 ml/kg/min. Bentall procedure or ascending aorta replacement was performed depending on conditions of aortic regurgitation. After the completion of distal and transverse anastomoses, systemic rewarming was started and the proximal reconstruction marked the completion of a standard CPB surgery.

### Statistical analysis

Data were analyzed with SPSS software (version 25.0 IBM Corp, Armonk, NY). Continuous variables were expressed as median and interquartile (IQR), the student *t*-test was applied for normally distributed continuous variables and Mann–Whitney *U* test for nonnormally distributed variables. Categorical variables were expressed as number and percentage of patients and analyzed with Chi-square or Fisher exact test, as appropriate. Logistic regression models were used to identify multivariable predictors for AKI. Considering the relatively low number of AKI patients (n = 51) in the cohort and in order to avoid overfitting in the model, the significant variables identified by univariable analysis were further evaluated in multivariable analysis. Receiver-operating characteristic (ROC) curves were constructed to assess the diagnostic potential of preoperative serum cystatin C (sCyC). The optimal cutoff was assessed by You-den’s index. Linear regression analysis was used to assess the correlation between the preoperative sCyC and sCr. Crude survival rate was estimated with Kaplan–Meier method. Differences in survival rate between groups were analyzed with log-rank tests. A *p* value of less than 0.05 was considered statistically significant.

## Results

### Demographic and clinical features

During screening period, 125 patients received emergency operation for TA-AAD. Among these patients, 1 required hemodialysis and 3 died within 24 h after operation were excluded from the study. As a result, 121 patients were enrolled in the study and 51 (42.1%) developed AKI.

Patient demographic and surgical features were summarized in Table 1 and Table 2. The median age of all enrolled patients was 34 years (IQR 30 to 36 years). Compared to patients who did not develop AKI, the prevalence of hypertension history and preoperative sCyC level was significantly higher in patients with AKI. In terms of surgical operative parameters, patients with AKI required less frequent intra-operative aortic valve management, while the proportion of total arch replacement (TAR) and the duration of deep hypothermic circulatory arrest (DHCA) were significantly increased in patients with AKI compared to patients without AKI.

**Table 1:** Comparison of preoperative variables

Variables	Total (*n* = 121)	AKI (*n* = 51)	Non-AKI (*n* = 70)	*P* Value^a^
Demographic data				
Age (year)	34.0 (30.0, 36.0)	35.0 (31.0, 37.0)	33.0 (30.0, 38.0)	0.204
Male (%)	99 (81.8)	40 (78.4)	59 (84.3)	0.410
BMI (kg/m2)	25.7 (22.0, 30.6)	26.3 (23.9, 32.1)	24.7 (20.1, 32.8)	0.091
Medical history				
Hypertension (%)	60 (49.6)	36 (70.6)	25 (35.7)	** < 0.001**
Diabetes mellitus (%)	1 (0.8)	1 (2.0)	0 (0)	0.421
Previous cardiac surgery (%)	6 (5.0)	3 (5.9)	3 (4.3)	0.696
Previous Coronary artery disease (%)	3 (2.5)	3 (5.9)	0 (0)	0.072
Cerebrovascular disease (%)	1 (0.8)	1 (2.0)	0 (0)	0.421
LVEF (%)	60.0 (56.0, 60.0)	58.5 (54.8, 60.0)	60.0 (57.0, 60.5)	0.257
Pericardial effusion (%)	1 (0.8)	0 (0)	1 (1.4)	1.000
Preoperative laboratory data				
WBC (109/L)	11.9 (9.1, 14.9)	12.2 (9.0, 15.9)	11.3 (9.2, 14.2)	0.171
sCr (μmol/L)	71.8 (56.7, 99.7)	80.0 (58.9, 103.0)	68.0 (53.9, 97.0)	0.099
sCyC	0.78 (0.55, 1.22)	1.21 (0.76, 1.67)	0.63 (0.49, 0.84)	** < 0.001**
PLT (109/L)	155.5 (108.8, 203.0)	165.0 (108.0, 208.0)	153.0 (109.5, 198.0)	0.669
ALB (g/L)	38.6 (35.1, 41.6)	38.0 (32.9, 41.2)	39.5 (35.4, 42.4)	0.120
Fibrinogen (g/L)	2.5 (2.0, 3.5)	2.4 (2.0, 3.5)	2.6 (1.8, 3.4)	0.998
Triglyceride (mmol/L)	1.2 (0.8, 1.7)	1.3 (0.9, 2.9)	1.2 (0.7, 1.5)	0.090
D-dimer (ng/mL)	3.1 (1.7, 5.2)	3.7 (2.2, 5.3)	2.9 (1.5, 5.1)	0.215

Data presented as n (%); median (IQR)

*Abbreviations*: *BMI* body mass index, *LVEF* left ventricular ejection fraction, *WBC* white blood cell, *SCr* serum creatinine, *SCyC* serum cystatin C, *PLT* platelet, *ALB* albumin

^a^*P* values indicate differences between AKI and Non-AKI. *P* < 0.05 was considered statistically significant

**Table 2:** Comparison of operative variables

Variables	Total (*n* = 121)	AKI (*n* = 51)	Non-AKI (*n* = 70)	*P* Value^a^
TAR (%)	75 (62.0)	40 (78.4)	35 (50.0)	**0.001**
CABG/MVR/MVP/TVP (%)	11 (9.1)	5 (9.8)	6 (8.6)	1.000
Aortic valve (%)	57 (47.1)	13 (25.5)	44 (62.9)	** < 0.001**
CPB time (min)	235.0 (203.0, 273.5)	239.5 (213.5, 306.0)	231.0 (197.3, 269.0)	0.075
Aortic cross-clamp time (min)	173.5 (138.0, 209.5)	175.0 (136.8, 222.3)	170.5 (138.0, 202.5)	0.558
DHCA time (min)	30.0 (19.5, 38.0)	33.0 (27.8, 40.3)	25.0 (16.0, 35.0)	**0.001**

Data presented as n (%); median (IQR)

*Abbreviations*: *TAR* total arch replacement, *CABG* coronary artery bypass graft, *MVR* mitral valve replacement, *MVP* mitral valvuloplasty, *TVP* tricuspid valvuloplasty, *CPB* cardiopulmonary bypass, *DHCA* deep hypothermic circulatory arrest

^a^*P* values indicate differences between AKI and Non-AKI. *P* < 0.05 was considered statistically significant

In-hospital outcomes were shown in Table 3. Postoperative mechanical ventilation duration, intensive care unit (ICU) and hospital stay were all prolonged in patients with AKI compared to patients without AKI. Unsurprisingly, postoperative hemodialysis and 30-day mortality were also increased in patients with AKI.

**Table 3:** Comparison of postoperative variables

Variables	Total (*n* = 121)	AKI (*n* = 51)	Non-AKI (*n* = 70)	*P* Value^a^
Drainage volume 24 h after surgery (ml)	540.0 (300.0, 907.5)	650.0 (292.5, 1256.3)	515.0 (307.5, 852.5)	0.288
Re-exploration for bleeding (%)	3 (2.5)	1 (2.0)	2 (2.9)	1.000
Dialysis (%)	15 (12.4)	15 (29.4)	0 (0)	** < 0.001**
Ventilation time (hour)	16.0 (12.0, 36.0)	29.5 (16.0, 64.0)	14.1 (9.9, 19.0)	** < 0.001**
Stroke (%)	12 (9.9)	3 (5.9)	9 (12.9)	0.205
Paraplegia (%)	3 (2.5)	0 (0)	3 (4.3)	0.262
Tracheostomy (%)	9 (7.4)	3 (5.9)	6 (8.6)	0.732
Deep sternal wound infection (%)	1 (0.8)	0 (0)	1 (1.4)	1.000
30-day mortality (%)	7 (5.8)	6 (11.8)	1 (1.4)	**0.041**
ICU Stay time (day)	4.0 (3.0, 7.0)	6.0 (4.0, 10.0)	3.5 (3.0, 5.0)	** < 0.001**
Hospital stay time (day)	20.0 (16.0, 26.5)	24.0 (17.0, 33.0)	19.0 (15.0, 25.0)	**0.006**

Data presented as n (%); median (IQR)

*Abbreviations*: *ICU* intensive care unit

^a^*P* values indicate differences between AKI and Non-AKI. *P* < 0.05 was considered statistically significant

Next, we examined variables mentioned above in the methods section by multivariable logistic regression assay and discovered that elevated preoperative sCyC (OR, 1.029; 95% CI, 1.007–1.051; *P* = 0.009) was identified as an independent risk factor associated with postoperative AKI (Table 4).

**Table 4:** Multivariable analysis of risk factors for acute kidney injury with KDIGO

Variable	OR	95% CI	*P* Value
Hypertension	0.857	0.231–3.178	0.818
sCyC	6.506	1.852–22.855	**0.003**
Aortic valve	0.310	0.078–1.226	0.095
TAR	1.465	0.352–6.093	0.599
DHCA time	1.024	0.973–1.078	0.818
Ventilation time	1.001	0.993–1.010	0.762

*Abbreviations*: *SCyC* serum cystatin C, *TAR* total arch replacement, *DHCA* deep hypothermic circulatory arrest, *OR* odds ratio, *CI* confidence interval

*P* < 0.05 was considered statistically significant

**Fig. 1:**
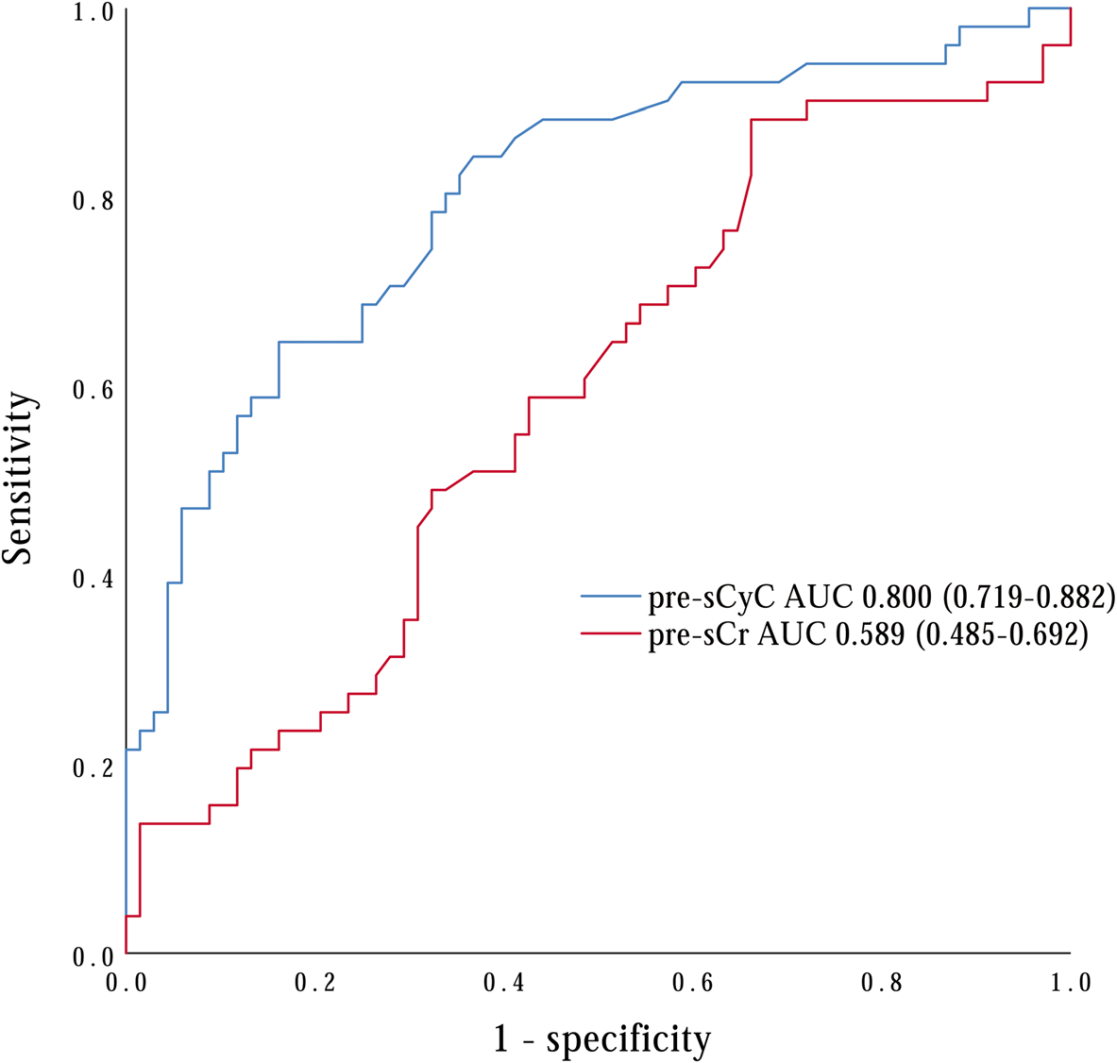
Receiver-operating characteristics of serum cystatin C and serum creatinine before the operation (AUC, areas under receiver-operating characteristic curve; pre-sCyC, preoperative serum cystatin C; pre-sCr, preoperative serum creatinine)

In addition, as presented in Fig. 1, we conducted ROC curve assay and calculated the areas under the ROC curve (AUC) of preoperative sCyC and sCr. The AUC of preoperative sCyC and sCr was 0.800 (95% CI: 0.719, 0.882; *P* < 0.001) and 0.589 (95% CI: 0.485, 0.692; *P* = 0.099) respectively, and the AUC of preoperative sCyC showed a higher value to predict postoperative AKI (*P* < 0.001). Our examination suggested that preoperative sCyC had a sensitivity of 64.7% and a specificity of 83.8% in predicting AKI with a diagnostic cut-off value of 0.895 mg/L.

**Fig. 2:**
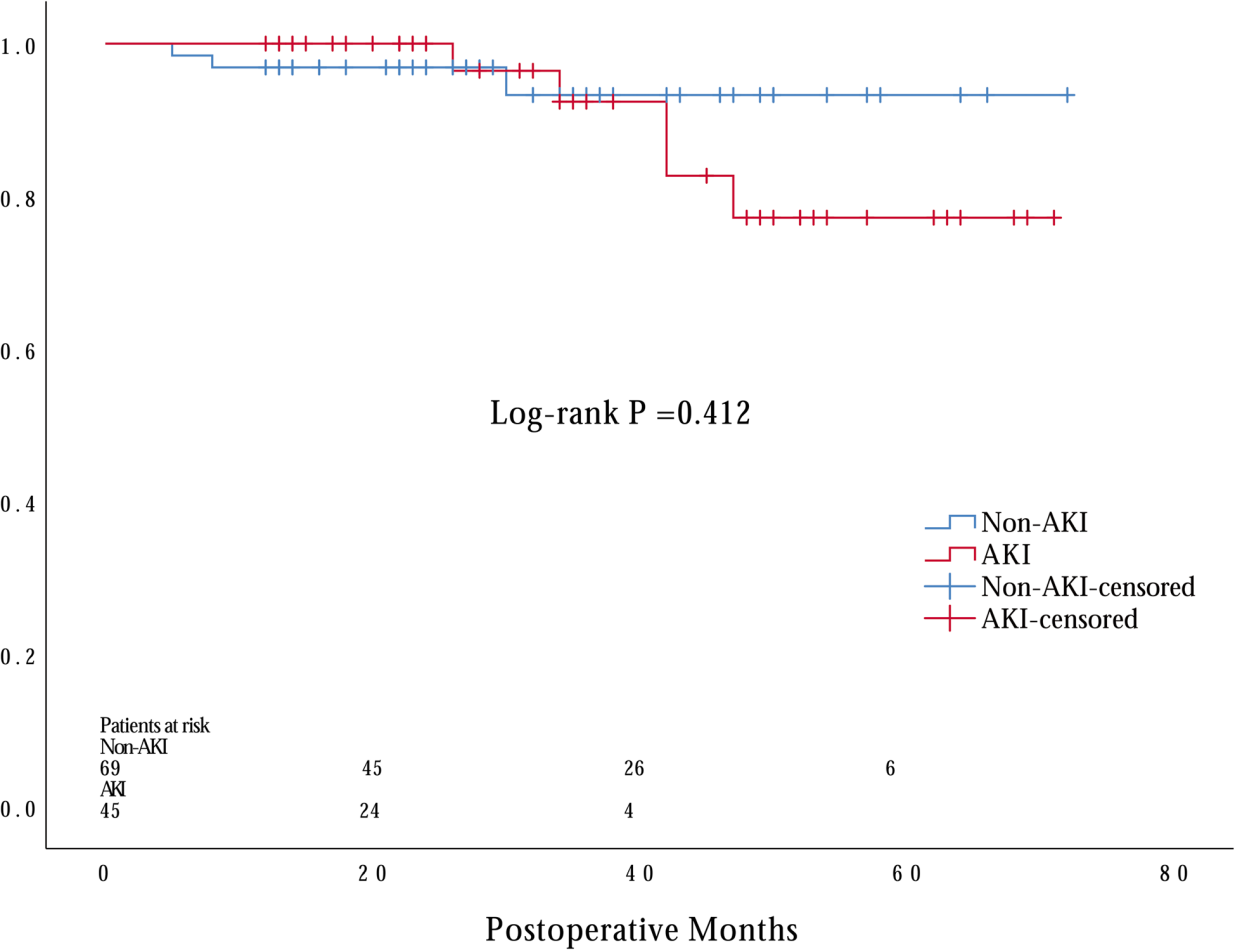
Kaplan–Meier estimate for late survival of young patients operated on for type A acute aortic dissection according to the presence of postoperative acute kidney injury

Unfortunately, 7 patients died during hospitalization. Among the remaining 114 patients, 5 patients that lost to follow-up and 1 patient who committed suicide 6 months after hospital discharge were excluded from the following survival analysis. The median follow-up period was 29 months. 5 patients in the AKI group and 3 patients in the non-AKI group died during follow-up period. There was no significant difference identified by Kaplan–Meier survival curves between two groups regarding long-term survival (Fig. 2; *p* = 0.412 by log-rank test).

## Discussion

To our knowledge, this was the first study to examine the incidence, risk factors and outcomes for AKI complication after TA-AAD emergency surgery in young patients. This study shows that postoperative AKI was associated with increased 30-day mortality rate in young patients who received emergency TA-AAD surgery. Multivariable logistic regression analysis and ROC curve assay demonstrated that elevated preoperative sCyC was an independent risk factor and might be considered as an diagnostic marker for postoperative AKI.

The incidence of postoperative AKI in our study was 42.1% and consistent to previous studies [1, 2], which suggested that postoperative AKI was a relative common complication in young patients. Similar to previous studies, 15 patients (12.4%) in this cohort required postoperative RRT, and all of whom belong to AKI group [8–10]. A previous meta-analysis suggested that patients with AKI had higher risk of experiencing chronic kidney disease and end-stage renal disease [11]. Despite significant advance in intensive care and renal replacement techniques have been achieved in recent years, the short- and long- term mortality of patients with AKI remains relatively high [12]. Our data suggested that young age was not a protective factor for postoperative AKI and cautions should be applied in preventing postoperative AKI in young patients.

The 30-day mortality of all enrolled patients was 5.8% (7 out of 121 patients), which was lower than previous studies which enrolled older patients [9, 13], indicating age as a significant risk factor affect 30-day mortality [14–16]. However, our data suggested a significant difference of 30-day mortality between patients with and without AKI. It was in consistent with previous studies suggesting postoperative AKI strongly influenced patients outcomes [1, 9, 17]. While regarding long-term mortality rate, we only observed a numerical difference but did not reach statistical significance between patients with and without postoperative AKI. This might due to the relatively small number of patients and insufficient follow-up time.

Logistic regression model suggested elevated preoperative sCyC as an independent risk factor for postoperative AKI. This discovery was consistent with previous study demonstrating elevated sCyC was an important trigger for AKI in patients underwent cardiac surgeries [18, 19]. While for TA-AAD surgical repair, there were few studies applied sCyC to elvaluate renal function. Another main finding of our study was the ability of preoperative sCyC in predicting persistent AKI with a high level of sensitivity (64.7%) and specificity (83.3%). The predictive ability was comparable to that reported by Parikh et al. [20] and Wang et al. [19], but much higher than one of our previous studies which enrolled both young and old patients [21]. This implied that sCyC had a better predictive ability in young patients. Some studies had shown that serum sCyC was a better indicator of kidney function compared to sCr [22, 23], and increased 24–48 h earlier than conventional clinical diagnosis of AKI could be made [24, 25]. As a well-accepted marker of renal function, sCr has several limitations and can be affected by postoperative hemodilution, muscle wasting, and malnutrition [26] which sometimes might result in overestimation of the kidney function. As sCyC does not secrete from tubular and excrete in urine solely through filtration, it has the potential to be a useful alternative marker for detecting both chronic and acute changes in glomerular filtration rate [27]. Our studies demonstrated that preoperative sCyC was an independent risk factor for postoperative AKI. And our data suggested that monitoring preoperative sCyC level might be helpful to identify patients who have increased risk of developing renal malfunction.

## Study limitations

This study has some limitations. Firstly, the relatively small number of patients enrolled in this study might have insufficient power to identify potential confounding baseline characteristics. Secondly, we only used sCr level for KDIGO classification which might have resulted in an underestimation of the incidence of AKI. Finally, because of the emergent nature of the disease, complete demographic data were absent for some patients.

## Conclusion

In conclusion, AKI after TA-AAD surgery was common in young patients and associated with increased short-term mortality. The elevated preoperative sCyC level was an independent risk factor for postoperative AKI and might be helpful in ensuring timely diagnosis.

## Data Availability

The datasets used or analyzed during the current study are available from the corresponding author on reasonable request.

## Abbreviations

AKI: Acute kidney injury
TA-AAD: Type A acute aortic dissection
KDIGO: Kidney disease improving global outcomes
RRT: Renal replacement treatment
sCyC: Serum cystatin C
AUC: Areas under the receiver-operating characteristic curve
AD: Aortic dissection
sCr: Serum creatinine
CPB: Cardiopulmonary bypass
IQR: Interquartile
ROC: Receiver-operating characteristic
TAR: Total arch replacement
DHCA: Deep hypothermic circulatory arrest
ICU: Intensive care unit
BMI: Body mass index
LVEF: Left ventricular ejection fraction
WBC: White blood cell
PLT: Platelet
ALB: Albumin
CABG: Coronary artery bypass graft
MVR: Mitral valve replacement
MVP: Mitral valvuloplasty
TVP: Tricuspid valvuloplasty
OR: Odds ratio
CI: Confidence interval
Pre-sCyC: Preoperative serum cystatin C
Pre-sCr: Preoperative serum creatinine
